# Pharmacokinetics of intravitreal antibiotics in endophthalmitis

**DOI:** 10.1186/s12348-014-0022-z

**Published:** 2014-09-10

**Authors:** Medikonda Radhika, Kopal Mithal, Abhishek Bawdekar, Vivek Dave, Animesh Jindal, Nidhi Relhan, Thomas Albini, Avinash Pathengay, Harry W Flynn

**Affiliations:** 1 Retina and Uveitis Department, L V Prasad Eye Institute, GMR Varalaxmi Campus, 11-113/1, Hanumantha waka Junction, Visakhapatnam 530040, Andhra Pradesh, India; 2 Srimati Kannuri Santhamma Centre for Vitreoretinal Diseases, L V Prasad Eye Institute, KAR Campus, Hyderabad 500034, Andhra Pradesh, India; 3 Department of Ophthalmology, Bascom Palmer Eye Institute, Miami 33136, FL, USA

**Keywords:** Intravitreal, Antibiotics, Pharmacokinetics, Endophthalmitis

## Abstract

Intravitreal antibiotics are the mainstay of treatment in the management of infectious endophthalmitis. Basic knowledge of the commonly used intravitreal antibiotics, which includes their pharmacokinetics, half-life, duration of action and clearance, is essential for elimination of intraocular infection without any iatrogenic adverse effect to the ocular tissue. Various drugs have been studied over the past century to achieve this goal. We performed a comprehensive review of the antibiotics which have been used for intravitreal route and the pharmacokinetic factors influencing the drug delivery and safety profile of these antibiotics. Using online resources like PubMed and Google Scholar, articles were reviewed. The articles were confined to the English language only. We present a broad overview of pharmacokinetic concepts fundamental for use of intravitreal antibiotics in endophthalmitis along with a tabulated compendium of the intravitreal antibiotics using available literature. Recent advances for increasing bioavailability of antibiotics to the posterior segment with the development of controlled drug delivery devices are also described.

## Review

### Introduction

Endophthalmitis whether exogenous or endogenous is anatomically and visually devastating for the patient and always presents a challenge to the treating physician. The vitreous is a transparent gelatinous avascular body, rich in collagen and hyaluronic acid, which provides a good culture medium for the microorganisms to proliferate. The presence of poorly developed local immune mechanisms also promotes microbial proliferation. For successful elimination of the infection in endophthalmitis, antibiotics must reach the intraocular space and adjacent ocular tissues. Static and dynamic ocular barriers which form part of the natural protective mechanisms of the eye impede the penetration of systemically and topically administered antibiotics. Satisfactory drug concentration in the vitreous can be achieved only by the intravitreal route. Over the years, intravitreal administration of antibiotics has become the mainstay of endophthalmitis management [1],[2]. In the absence of adequate antimicrobial concentrations, irreversible tissue destruction ensues [3].

Various factors are responsible for the poor penetration of topical and systemic antibiotics in the vitreous. Baring a few exceptions like systemically administered fluoroquinolones and linezolid [3]–[5], topical and systemic antibiotics do not achieve adequate therapeutic levels due to various physiological barriers. Topically instilled medicines are diluted by the tear film, causing loss of significant drug in the lacrimal flow [6]. Further low molecular weight antibiotics also undergo systemic absorption from the conjunctival capillaries and the nasolacrimal mucosal surfaces, leading to further drop in bioavailability [7]. The corneal epithelium also has tight junctions, leading to poor paracellular drug penetration especially for ionic drugs [8]. The posterior barrier between the bloodstream and the eye is comprised of retinal pigment epithelium (RPE) and the tight walls of retinal capillaries. Unlike retinal capillaries, the vasculature of the choroid has extensive blood flow and leaky walls. Systemically administered drugs easily gain access to the choroidal extravascular space, but thereafter, distribution into the intraocular space via the retina is limited by the RPE and the retinal endothelium [9]. Thus, intravitreal administration serves as the only direct access to the vitreous cavity by bypassing the blood retinal barrier and achieving higher concentrations of drugs for prolonged periods of time [10].

Using online resources like PubMed and Google Scholar, articles of the antibiotics which have been used for intravitreal route and the pharmacokinetic factors influencing the drug delivery and safety profile of these antibiotics were reviewed. The articles were limited to the English language only. The keywords searched were endophthalmitis, intravitreal antibiotics and pharmacokinetics.

### History of intravitreal antibiotics

Experimental studies for treatment of endophthalmitis in rabbit eyes with intraocular antibiotics like penicillin and sulphonamides were reported as early as the 1940s [11]. Intravitreal penicillin was found to have a favourable though limited effect on traumatic endophthalmitis in these studies. In the 1970s, Peyman and associates reported the safety and efficacy of various intravitreal antibiotics in experimentally induced endophthalmitis in rabbit eyes and established the recommended doses of various intravitreal antibiotics in human eyes [12],[13]. Favourable results of treatment of acute postoperative endophthalmitis with intravitreal antibiotics - vancomycin for staphylococcal endophthalmitis and aminoglycosides for Gram-negative endophthalmitis - were reported during the 1970s [13]–[15]. However, as the macular toxicity of aminoglycoside antibiotics became known, ceftazidime, a third-generation cephalosporin, has become the preferred alternative [16]. In recent times, alternate antibiotics like intravitreal piperacillin-tazobactam have been studied both in animal models and clinically especially in cases of *Enterobacter* species and multidrug-resistant *Pseudomonas* endophthalmitis with favourable outcomes and have emerged as a useful alternative to ceftazidime [17]–[19].

### Factors influencing antibiotic pharmacokinetics

Intravitreal injection of antibiotics bypasses the various anatomical and physiological ocular barriers. The drug diffuses freely in the vitreous cavity and reaches the retinal surface, facilitated by extraocular movements [20]. However, the drug distribution and clearance from the vitreous are influenced by various factors including ionic nature, molecular weight of the drug molecule, surgical status and effect of ocular inflammation. In order to achieve a sustained therapeutic drug concentration in the vitreous, the frequency of administration should be based on the half-life (*t*_1/2_). The elimination of drug usually follows first-order kinetics and is proportional to the amount of drug available and the volume of the vitreous. Factors influencing the pharmacokinetics of intravitreal antibiotics have been described below briefly [21].

1. Route of exit: Drug molecules can leave the eye through the anterior route or the posterior route. Large molecules are known to leave the eye predominantly by the passive diffusion across the vitreous to the anterior chamber and through Schlemm's canal. These include vancomycin, aminoglycosides, macrolides and rifampicin. The posterior route is achieved by active transport in the capillaries and the retinal pigment epithelium through which smaller drug molecules like beta-lactams, clindamycin and fluoroquinolones are cleared [22].

2. Ionic nature: Cationic drugs like vancomycin, aminoglycosides, erythromycin and rifampicin undergo clearance by passive diffusion into the aqueous and leave the eye via the anterior chamber with a *t*_1/2_ of about 24 h [23]–[25]. Anionic drugs like beta-lactams, cephalosporins and clindamycin primarily undergo clearance more rapidly across the blood retinal barrier via the posterior route and exit the eye via uveal blood flow [23],[24]. This is facilitated by active transport by the retinal pigment layer pump. Hence, they have shorter *t*_1/2_ of about 8 h. Fluoroquinolones which are zwitterions are cleared via both routes and hence have the shortest *t*_1/2_[26],[27].

3. Solubility coefficient of the drug: Lipophilic antibiotics like fluoroquinolones and chloramphenicol can be transported by passive diffusion, while water-soluble antibiotics like beta-lactams leave the eye via active transport [23],[24].

4. Status of ocular inflammation: In a non-inflamed eye, the anterior route is poorly efficient, and hence, antibiotics (vancomycin, aminoglycosides, erythromycin and rifampicin) eliminated by this route show long half-life values. Thus, drugs eliminated through the anterior route have a faster clearance in an inflamed eye [25]. For drugs mainly eliminated by the posterior route (beta-lactams, cephalosporins and clindamycin) in the case of an inflamed eye, the drug clearance is retarded due to compromise of the retinal pigment epithelial (RPE) pump or the active transport. Thus, their half-life is extended [24],[28]–[30].

5. Surgical status of the eye: Clearance of antibiotics which leave the eye through the anterior route is more rapid in aphakic eyes, while those which leave the eye primarily through the posterior route are cleared more rapidly in vitrectomized eyes. Hegazy et al. demonstrated retinal toxicity to routinely used doses of intravitreal antibiotics in silicone oil-filled eyes. Retinal toxicity was hypothesized due to reduction of the preretinal space; the drug is confined to the limited aqueous-filled space surrounding the oil bubble and has a longer elimination time. They recommended using one quarter of the recommended dose to prevent retinal toxicity [31].

6. Molecular weight: It has been found that the retention of the drug in the vitreous cavity increases with increase in relative impermeability of the retina. As most drugs have a molecular weight of <500 Da, the half-life is less than 72 h, requiring repeat injection at that interval depending on the clinical situation [29].

7. Vitreous liquefaction: If vitreous liquefaction occurs in the anterior few millimetres and the posterior few millimetres of the globe, it can lead to the quick egress of the drug out of the eye, leading to shortening of its half-life [29].

8. Solution density: If the density of the injected solution is greater than water, it may settle down under gravity and cause localized toxicity. This may require intermittent repositioning of the patient's head to avoid such an eventuality [32].

9. Frequency of intravitreal antibiotic administration: The parameters deciding the frequency of repeat administration of antibiotics are clinical response, half-life, drug clearance from the eye and surgical status of the eye. The aim of repeat dosing should be to optimize the duration of drug exposure to concentrations above the minimum inhibitory concentration (MIC), rather than to aim at higher peak levels. Adequate and safe antibiotic levels can be better achieved by more frequent rather than higher dosages [28].

### Intravitreal antibiotics: dosing and frequency

Table 1 is a pooled compendium of all published information pertaining to the dosing of antibiotics studied and used for intraocular use in treating experimental endophthalmitis and human eyes [16]–[59].

**Table 1:** Pharmacokinetics of intravitreal antibiotics - recommended dosing and frequency of administration

**Serial number**	**Drug**	**Model**	**Recommended dose (μg/0.1 ml)**	**Route of clearance**	**Half-life (*t***_**1/2**_**) in vitreous**			**Frequency of repeat injections (h)**	**Susceptible microorganisms**
					**Non-inflamed phakic eyes**	**Inflamed eyes**	**Aphakic vitrectomized eyes**		
1.	Amikacin [16],[33]–[35]	Human	400	Anterior	NA	NA	NA	24 to 48	Aerobic GNBs, *Pseudomonas aeruginosa*
		Rabbit	400		25.5 h	<24 h	7 h	24 to 48	
2.	Ampicillin [36]	Human	5,000	Posterior	NA	NA	NA	48	GPC, enterobacteria, therapeutic option for infections caused by MDR pathogens
3.	Amphotericin-B [37]	Human	5 to 10	Posterior	8.9 days	NA	1.8 h	NA	Yeasts, filamentous fungi (resistance reported for various species of *Aspergillus*)
		Rabbit	10		4.7 days	NA	NA	NA	
4.	Aztreonam [38]	Rabbit	100	Posterior	7.5 h	NA	NA	12	Excellent activity against family *Enterobacteriaceae*; moderate activity against *Pseudomonas*
5.	Carbenicillin [24]	Rabbit	2,000	Anterior	5 h	NA	NA	15 to 24	*Pseudomonas*, therapeutic option for infections caused by MDR pathogens
		Monkey	1,000		10 h	NA	NA	NA	
6.	Cephazolin [24],[39]	Human	2,250	Posterior	6.5 h	10.5 h	NA	24	GPC, GPB, *E. coli*, *Proteus*, *H. influenza*
		Rabbit	2,250		6.5 h	10.4 h	6 h	NA	
7.	Ceftazidime [16],[40]	Human	2,250	Both posterior and anterior	NA	NA	NA	48 to 72	Aerobic GNBs, GPBs including *Pseudomonas*
		Rabbit	2,250		13.8 h	10.1 h	4.7 h	72	
8.	Ceftriaxone [21],[29]	Rabbit	2,000	Both posterior and anterior	NA	NA	NA	48 to 72	Aerobic GNBs
9.	Cefuroxime [21],[29]	Human eyes	1,000	Posterior	NA	NA	NA	48 to 72	GPC, GPB, GNC, GNB including *Pseudomonas aeruginosa*, penicillinase-producing *N. gonorrhoeae*, ampicillin-resistant *H. influenzae*
10.	Ciprofloxacin [26],[28]	Human	100	Both anterior and posterior	3.5 to 5.5 h	NA	1.2 h	12	Broad-spectrum activity against aerobic Gram-positive and Gram-negative bacteria, *Actinomyces*, *Nocardia* spp.
		Rabbit	100		2.2 h	NA	NA	NA	
11.	Clarithromycin [41]	Rabbit	<1,000	Posterior	2 h	NA	NA	NA	GPC, GPB, *Chlamydia*, *Toxoplasma gondii*
12.	Clindamycin [42]	Human	1,000	Posterior	40 h	NA	NA	72	GPCs - staphylococci, pneumococci; GPBs - *Bacillus*; GNBs - *Bacteroides*, *Fusobacterium*; resistance - enterococci, *Enterobacteriaceae*, *Clostridium*, *Toxoplasma gondii*
13.	Chloramphenicol [43]	Human	2,000	Posterior	NA	NA	NA	24	Gram-negative bacteria, *Rickettsia*, *Borellia recurrentis*; moderately active against Gram-positive bacteria and *Mycobacterium tuberculosis*
14.	Daptomycin [44]	Rabbit	200	Posterior	42 h	NA	NA	Single dose	Gram-positive organisms, MRSA, VRSA, pneumococci, enterococci
15.	Dalfopristine/quinopristine [45]	Rabbit	400	Posterior	NA	NA	NA	48	Active against VRSA
16.	Doxycycline [46]	Rabbit	125	NA	NA	NA	NA	NA	Broad-spectrum - Gram-positive and Gram-negative bacteria, *Spirochaetes*, *Rickettsia*, *Chlamydiae*, *Mycoplasma*, *Actinomyces*, *Entamoeba histolytica*, atypical mycobacteria
17.	Fluconazole [47]	Rabbit	200	Posterior	3.08 h	NA	NA	NA	Yeasts
18.	Gentamicin [48],[49]	Human	200	Anterior	40 to 60 h	20 to 40 h	<40 h	72 to 96	Aerobic GNBs
		Rabbit	40 to 70		32 h	19 h	12 h	NA	
19.	Imipenem [50]	Rabbit	50 to 100	Posterior	NA	NA	NA	NA	MDR GPB, GNBs including *Psedomonas aeruginosa*, therapeutic option for infections caused by MDR pathogens
20.	Linezolid [5],[51]	Rabbit	400	NA	2 h	NA	NA	NA	Aerobic GPC including MRSA and vancomycin-resistant enterococci
21.	Moxifloxacin [52]	Rabbit	200	Both anterior and posterior	1.72 h	Prolonged	NA	12	Broad-spectrum activity against Gram-positive and Gram-negative organisms
22.	Ofloxacin [27]	Rabbit	200 to 500	Both anterior and posterior	5.6 h	9.7 h	NA	24	Broad-spectrum activity against Gram-positive and Gram-negative organisms
23.	Penicillin [11]	Human	2 to 4,000 units	Posterior	NA	NA	NA	48	Broad-spectrum activity against Gram-positive organisms, *Spirochaetes*
24.	Piperacillin/tazobactam [17]–[19]	Human	225	Posterior	NA	NA	NA	NA	Effective GNBs, *Staphylococcus epidermidis* and *Pseudomonas aeruginosa*; therapeutic option for infections caused by MDR pathogens
		Rabbit	<250		NA	NA	NA	NA	
25.	Sulfamethoxazole/trimethoprim [52]	Rabbit	1,600 trimethoprim	Anterior	NA	NA	NA	NA	Broad-spectrum antibacterial activity; *Toxoplasma gondii*
26.	Tobramycin [53]	Human	200 to 400	Anterior	NA	NA	NA	NA	Aerobic Gram-negative organisms
		Rabbit	750		NA	NA	NA	72 to 96	
27.	Trovafloxacin [54]	Rabbit	25	Both anterior and posterior	NA	NA	NA	24 to 48	Expanded spectrum against Gram-positive and Gram-negative bacteria
28.	Vancomycin [25],[55]–[57]	Human	1,000	Anterior	25.5 to 56 h	48 h	9.8 h	72	Active against GPCs - MRSA and MDR *Staphylococcus epidermidis*
		Rabbit			25.1 h	NA	8.9 h	NA	
29.	Voriconazole [58]	Human	50 to 200	Posterior	2.5 to 6.5 h	NA	NA	NA	Broad-spectrum activity against moulds and yeasts
		Rabbit	25		2.5 h	NA	NA	NA	
30.	Meropenem [73]	Human		Posterior	2.6 h	NA	NA	NA	*Pseudomonas*, *Bacteroides*, *Clostridia*, *Listeria*, *Enterobacteriaceae*
		Rabbit	0.5						

GPC, Gram-positive cocci; GPB, Gram-positive bacilli; GNB, Gram-negative bacilli, GNC, Gram-negative cocci; MDR, multidrug-resistant; MRSA, methicillin-resistant *Staphylococcus aureus*; VRSA, vancomycin-resistant *Staphylococcus aureus*; NA, not available.

### Preparation of intravitreal antibiotics

Since the recommended therapeutic dosage of intravitreal antibiotics is very small and carefully titrated to prevent retinal toxicity, it is important that this dose is maintained each time an injection is prepared [39]. Standard protocols have to be followed to ensure accuracy, precision as well as reproducibility. The injections have to be prepared under strict aseptic conditions, under a certified laminar flow by trained personnel. Preferably, a printed reference display sheet should be consulted while preparing injections every time as dilution errors may be toxic. Mehta et al. reported that vancomycin, ceftazidime and moxifloxacin prepared in single-use polypropylene syringes retain potency, sterility and stability up to 24 weeks when stored at −20°C or −80°C [60].

### Antibiotic resistance

Indiscriminate and injudicious use and abuse of antibiotics have led to the development of resistant bacterial strains. These include the ocular and nasopharyngeal flora as well as pathogenic organisms like those causing keratitis and other ocular infections. Endophthalmitis caused by these organisms is associated with more severe clinical course and worse visual outcomes [60]–[62]. This problem of emergence of resistance to standard antibiotic therapy has forced clinicians to continually evaluate the best intraocular antibiotics available for the treatment of bacterial endophthalmitis. In such situations, the choice of antibiotics is judiciously guided by culture results and sensitivity patterns of the causative organism. However, it is also known that *in vitro* resistance need not be mirrored with *in vivo* sensitivity and routinely administered antibiotic doses provide intraocular drug concentrations which are much higher than the MICs of most pathogens [61],[62]. Knowledge of pharmacokinetics, susceptibility patterns and minimum inhibitory concentration serves to properly predict the *in vivo* efficacy of antibiotics against target pathogens [62].

### Combination therapy

Combination intravitreal therapy is used often in polymicrobial cases or in empirical treatment of endophthalmitis [74]. The physicochemical properties of the various drugs used for combined injections should be well known by the physician as they form the basis of possible adverse drug interactions. The two most common physicochemical entities that can cause adverse drug interactions are dilution-dependent reactions and acid-base reactions. Adverse reactions when they occur become evident by physical changes like precipitates, effervescence, haziness and viscosity changes. Precipitates are avoided by injecting the drugs through different syringes. Still, the development of subclinical microprecipitates cannot be ruled out. It has been reported that such precipitate formation may still allow enough antibacterial activity of the drug at intravitreal concentrations to be therapeutically active [75]. Changes in individual drug half-life post multiple injection have not been studied in literature.

### Future trends

Advances in ocular drug delivery system research are expected to provide new tools for the treatment of posterior segment diseases, providing improved drug penetration, prolonged action, higher efficacy, improved safety and less invasive administration, resulting in higher patient compliance. Various attempts have been made to improve drug bioavailability by increasing both drug retention and drug penetration. Patient compliance and comfort considerations in drug administration are very important factors that may impact the drug therapeutic efficacy [64]. These attempts can be divided into two main categories: bioavailability improvement and controlled release drug delivery. The first category includes gels, emulsions, viscosity enhancers, pro-drugs, liposomes and iontophoresis. The second category includes various types of polymeric inserts, implants and nanoparticles.

A pro-drug is defined as an inactive species obtained by chemical modifications of the active drug which, when delivered, will release the active drug essentially in a single step (i.e. enzymatic conversion). Usually, ophthalmic pro-drugs are lipophilic esters or diesters with better permeability than the parent compound. Lipophilicity increases uptake of the pro-drug across lipophilic membranes which otherwise act as a barrier to hydrophilic drugs. If the drug is incorporated into a polymeric vehicle which controls the release of the pro-drug, a sustained delivery of the drug to the retina and vitreous layers may be possible [65].

Liposomes are vesicles composed of one or more phospholipid bilayers separated by aqueous compartments. Liposomes can encapsulate hydrophilic drugs in the aqueous cavity or introduce hydrophobic drugs into the membrane as a component. They act as reservoir-type carriers and possess qualities which can make them ideal for certain posterior segment uses [66],[67]. Intravitreally administered liposomal systems could both significantly increase drug half-life and minimize the intraocular side effects of drugs used (i.e. ganciclovir and 5-fluorouridine). Intravitreal injection of liposomes containing a lipid pro-drug of ganciclovir inhibited CMV retinitis in rabbits [68],[69].

The mechanism of iontophoresis involves applying an electrical current to an ionisable substance to increase its mobility across a surface. The EyeGate II Delivery System (EGDS; EyeGate Pharmaceuticals, Inc., Waltham, MA, USA), a novel iontophoretic system, has been designed to achieve optimal therapeutic levels of drug in the anterior and posterior segments of the eye, while simultaneously minimizing systemic distribution [70]. The system consists of an inert electrode which electrolyzes water to produce hydronium ions. These hydronium ions propel charged drug molecules. Studies demonstrating safety and efficacy profile show promise for the future [71].

Nanoparticles are defined as particles with a diameter of less than 1 nm (10^−9^ m) consisting of various biodegradable materials, such as natural or synthetic polymers, lipids, phospholipids and metals. Studies have shown that nanoparticles of different sizes and electric charges, when injected into the vitreous, migrate through the retinal layers and tend to accumulate in the retinal pigment epithelium (RPE) cells up to 4 months after a single intravenous injection [72].

Pharmacokinetics, safety and efficacy of newer antibiotics and antifungals need to be continually explored and established in view of the emerging multidrug and sometimes pan-drug resistance amongst organisms causing systemic and ocular infections [62]. For sustained drug delivery and minimizing chances of retinal toxicity, intravitreal drug effects of delivering drugs in liposomes or microspheres have been studied which could provide therapeutic drug levels for up to a month [63],[64]. Non-biodegradable and biodegradable devices or implants have been investigated [65]–[67]. Utility of pro-drugs, permeability enhancers, particulate drug delivery systems xand iontophoresis is currently being explored for sustained intraocular drug delivery [66],[67].

## Conclusions

The management of infectious endophthalmitis has evolved from the usage of systemic antibiotics in the past to the current use of intravitreal antibiotics, paving the way for nanotechnology in drug delivery in the future. Successful management of endophthalmitis could be enhanced by better understanding of pharmacokinetics of intravitreal antibiotics. Emergence of drug resistance amongst bacteria remains a matter of concern.

## Competing interests

The authors declare that they have no competing interests.

## Authors' contributions

MR, KM and AB were involved in the preparation of the manuscript. MR, KM, AB, VD, AJ and NR were involved in the collection of data. TA, AP and HWFJ corrected the manuscript. All authors read and approved the final manuscript.
